# Mass spectrometry-based proteomic profiling of human tauopathy brains suggests mitochondria-associated alterations

**DOI:** 10.3389/fnmol.2026.1815858

**Published:** 2026-05-22

**Authors:** Alfi Raudatil Jannah, Mai Hasegawa, Jonathan Ham, Norikazu Hara, Tamao Tsukie, Ai Obinata, Masataka Kikuchi, Kensaku Kasuga, Haruyasu Yamaguchi, Hideomi Hamasaki, Mari Tada, Akiyoshi Kakita, Masaki Matsumoto, Akinori Miyashita, Takeshi Ikeuchi

**Affiliations:** 1 Department of Molecular Genetics, Brain Research Institute, Niigata University, Niigata, Japan; 2 Department of Pathology, Brain Research Institute, Niigata University, Niigata, Japan; 3 Center for Human Brain Resource Initiative (ChBRI), Niigata University, Niigata, Japan; 4 Gunma University, Maebashi, Japan; 5 Department of Omics and Systems Biology, Graduate School of Medical and Dental Sciences, Niigata University, Niigata, Japan

**Keywords:** Alzheimer’s disease, corticobasal degeneration, mass spectrometry, progressive supranuclear palsy, snRNA-seq, tau, tauopathies, tauopathy

## Abstract

Tauopathies are neurodegenerative disorders characterized by intracellular accumulation of abnormal tau encoded by *MAPT*, yet their molecular mechanisms remain incompletely understood. We aimed to identify proteomic signatures associated with the primary tauopathies corticobasal degeneration (CBD) and progressive supranuclear palsy (PSP), as well as the secondary tauopathy Alzheimer’s disease (AD), and to characterize their interaction networks. Total homogenates from the specified cortical region of postmortem brains of AD (*n* = 4), CBD (*n* = 4), PSP (*n* = 4), and control (*n* = 4) subjects were analyzed by mass spectrometry (MS). Differentially expressed proteins were subjected to functional enrichment and protein–protein interaction (PPI) network analyses. Reproducibility was assessed using JESS-based western blotting (WB), and selected candidates were examined by immunohistochemistry (IHC) and single-nucleus RNA sequencing (snRNA-seq) to evaluate cell-type–specific transcriptomic profiles. In the four-group comparison, 859, 114, 6, and 1 proteins showed *P*_*FDR*_ < 0.05, < 0.01, < 0.005, and < 0.001, respectively. Six proteins (AK3, ATP5PD, COX7C, PPA1, PREP, and UQCRC1) with *P*_*FDR*_ < 0.005 formed a highly interconnected network enriched for mitochondrial pathways, including oxidative phosphorylation and respiratory electron transport. WB validation showed strong concordance for four proteins (AK3, PREP, PPA1, and ATP5PD). IHC confirmed neuronal expression of PPA1 and PREP and revealed prominent microglial PPA1 immunoreactivity in PSP brains. snRNA-seq provided complementary cell-type–specific transcriptomic alterations. These findings suggest mitochondria-associated molecular changes shared across primary and secondary tauopathies; however, given the exploratory nature of this study, these observations should be interpreted cautiously and considered hypothesis-generating, warranting further investigation.

## Introduction

Tauopathies are a group of neurodegenerative disorders characterized by the abnormal aggregation and deposition of hyperphosphorylated tau protein in the brain. Despite this shared feature, they exhibit marked heterogeneity in clinical and pathological characteristics (Zhang et al., 2022). Alzheimer’s disease (AD), the most common secondary tauopathy, is characterized by amyloid-β plaques and neurofibrillary tangles composed of mixed three-repeat (3R) and four-repeat (4R) tau isoforms, whereas corticobasal degeneration (CBD) and progressive supranuclear palsy (PSP) are primary 4R tauopathies distinguished by astrocytic plaques, tufted astrocytes, and coiled bodies. Despite these well-established neuropathological features, clinical differentiation remains difficult due to overlapping symptoms, and validated biomarkers are still largely limited to AD. Therefore, identifying disease-specific molecular signatures remains an important goal.

Diversity in tau isoforms and post-translational modifications (PTMs) contributes to this heterogeneity (Yang et al., 2024). Six tau isoforms are expressed in the adult human brain, consisting of a balanced mixture of 3R and 4R tau. This equilibrium is disrupted in disease: AD accumulates both isoforms, whereas PSP and CBD predominantly accumulate 4R tau and Pick’s disease primarily accumulates 3R tau. PTMs such as phosphorylation and truncation further modulate tau aggregation propensity, and recent cryo-electron microscopy studies have revealed distinct tau filament conformers characteristic of each tauopathy (Vaquer-Alicea et al., 2021).

Mass spectrometry (MS)-based proteomics provides an unbiased strategy to investigate these molecular differences. Prior studies have frequently analyzed soluble and insoluble fractions separately, uncovering disease-specific patterns in phosphorylation, aggregation, and solubility (Kavanagh et al., 2025; Lantero-Rodriguez et al., 2024; Nementzik et al., 2025). Although informative, these fractionation approaches capture only part of the proteome and may introduce additional variability.

In contrast, analysis of total homogenates from the specified brain region (hereafter referred to simply as “total homogenates”) preserves the full proteomic landscape, encompassing both soluble proteins and insoluble aggregates (Johnson et al., 2024). This approach simplifies sample processing, enhances reproducibility, and enables direct molecular comparisons across diseases. Although dilution of low-abundance proteins is a recognized limitation, total homogenates provide a holistic view of disease-associated molecular changes and are well suited for exploratory characterization.

Here, we applied high-resolution MS to total homogenates from 16 postmortem brains, including AD (*n* = 4), CBD (*n* = 4), PSP (*n* = 4), and control (*n* = 4) subjects. Our aim was to identify disease-characterizing proteins, including tau isoforms and their PTM signatures, as well as non-tau proteins. By defining these proteomic profiles, we seek to clarify mechanisms underlying tauopathy heterogeneity and provide a basis for biomarker discovery and therapeutic development. Although the sample size is modest, this exploratory study is intended to generate hypotheses and guide future large-scale investigations.

## Subjects and methods

An overview of the analytical workflow of this study is shown in Supplementary Figure 1.

### Subjects used in the study

We analyzed a total of 16 human postmortem brains, consisting of four cases each neuropathologically diagnosed as AD, CBD, and PSP, along with four controls that showed no characteristic neuropathological abnormalities. These samples were used for MS, western blotting (WB), immunohistochemistry (IHC), and single-nucleus RNA sequencing (snRNA-seq) analyses described below. Demographics of the subjects used in this study are provided in Supplementary Table 1.

### LC-MS/MS

Total homogenates were prepared from each postmortem brain sample (approximately 100 mg) according to the method described by Diner et al. (2017). Each homogenate containing 100 μg of protein was used as the starting material. Proteins were precipitated with trichloroacetic acid (TCA), followed by acetone washing, air-drying, and resuspension in 100 mM ammonium bicarbonate based on the estimated protein concentration. After TCA precipitation, proteins were digested with trypsin, and cysteine residues were subsequently reduced and alkylated. Peptides corresponding to 2.5 μg of protein were then purified using SDB-XC StageTips (3M).

All samples were analyzed on a TripleTOF 5600+ mass spectrometer (SCIEX) coupled to an Ekspert nanoLC 415 system operated in direct-injection mode. Peptides were dissolved in 12.5 μL of 0.1% trifluoroacetic acid, and 1 μL of each sample was injected into the nanoLC system. Peptide separation was performed on an in-house-packed 15 cm column containing 2 μm octadecyl silane particles (Chemicals Evaluation and Research Institute) using a linear gradient of 5–32% solvent B for 60 min, followed by 32–90% B for 5 min and 90% B for 5 min, at a flow rate of 300 nL/min. Solvent A consisted of 0.1% formic acid in water, and solvent B consisted of 0.1% formic acid in acetonitrile.

Data acquisition was performed in SWATH mode. MS parameters were as follows: ion source gas 1, 20; ion source gas 2, 0; curtain gas, 10; interface heater temperature, 150°C; ion spray voltage floating, 2,300 V; and declustering potential, 80 V. MS1 spectra were acquired from 380 to 1,400 m/z with a 50 ms accumulation time. MS2 spectra were collected from 200 to 1,600 m/z in high-sensitivity mode with a 100 ms accumulation time. The collision energy was set to rolling collision energy with a collision energy spread of 15. Thirty-two SWATH windows (12.5 Da wide) were acquired across the 480–880 m/z range with a 1 Da overlap.

### Data processing

All raw data were converted to dia files and analyzed using a predicted spectral library, generated in library-free search mode against human “reviewed” UniProt sequences (downloaded from UniProt on December 24, 2024), with DIA-NN (version 1.9.2) (Demichev et al., 2020). The search parameters were configured as follows: enzyme specificity was set to trypsin, allowing one missed cleavage; carbamidomethylation of cysteine was designated as a fixed modification; Oxidation of methionine was specified as variable modifications; the maximum number of variable modifications was set to 1; quantification strategy was configured as QuantUMS (high precision); and the match-between-runs feature was enabled.

### PPI network analysis

To investigate functional relationships among proteins identified by MS, we constructed a PPI network using the STRING database^1^ (Szklarczyk et al., 2023). The list of MS-identified proteins was uploaded, and interaction networks were generated from known and predicted associations, including experimental evidence, curated databases, co-expression, and computational inference. A minimum required interaction score was set to high confidence (0.700) to limit false-positive interactions. The resulting network was visualized to assess connectivity and functional clustering.

### snRNA-seq

snRNA-seq was performed on isolated nuclei obtained from the same 16 postmortem brains (Supplementary Table 1), following the procedure described by Etani et al. (2025). Briefly, nuclei were isolated from ∼20 mg of fresh-frozen brain tissue using the Minute Single Nucleus Isolation Kit for Neuronal Tissues/Cells (Invent Biotechnologies), stained with ReadyCount Stains (Thermo Fisher Scientific), and quantified using a Countess II FL Automated Cell Counter (Thermo Fisher Scientific). Approximately 10,000 nuclei per sample were processed using the Chromium Next GEM Single Cell 3’ Kit v3.1 on a Chromium Controller (10x Genomics), followed by reverse transcription, cDNA amplification, and library preparation. Libraries were sequenced on an Illumina NextSeq 2000 platform. Sequencing data were processed with Cell Ranger (v7.0.0) for demultiplexing, alignment to GRCh38-2020-A, and gene counting. Downstream analyses were conducted in R using Seurat (v5.0.1). Low-quality nuclei (<200 or > 14,000 genes; > 5% mitochondrial transcripts) were excluded, data were normalized using SCTransform, and doublets were removed. Dimensionality reduction, clustering, and uniform manifold approximation and projection (UMAP) visualization were performed. For the snRNA-seq analysis, differential gene expression was assessed using MAST, and a Bonferroni-adjusted *P* < 0.01 was considered statistically significant.

### WB

We performed WB using the JESS automated capillary-based immunoassay system (ProteinSimple), according to the manufacturer’s instructions. Total homogenates prepared from the same 16 postmortem brain tissues (Supplementary Table 1) were used for analysis. Protein concentrations were determined in advance, and equal amounts of protein were loaded for each sample. Samples were mixed with fluorescent master mix and denatured at 95°C for 5 min prior to loading. Protein separation and immobilization were carried out automatically within capillaries based on size, followed by incubation with primary antibodies. The primary antibodies used in this study are listed in Supplementary Table 9, including their sources and dilution conditions. After incubation with horseradish peroxidase conjugated secondary antibodies, chemiluminescent detection was performed within the instrument. Signal detection, data acquisition, and quantification were conducted using the Compass for SW software (ProteinSimple). Protein expression levels were normalized to total protein signals to ensure comparability across samples. All procedures were performed under identical conditions to minimize technical variability. The use of this automated capillary-based system enables highly reproducible and quantitative protein detection compared with conventional gel-based WB.

### IHC

IHC was performed on 4-μm-thick sections from formalin-fixed, paraffin-embedded frontal lobe tissue blocks obtained from the same 16 subjects listed in Supplementary Table 1, as described previously (Takeuchi et al., 2016), using the antibodies listed in Supplementary Table 9. As detailed in Supplementary Table 1, these blocks included tissues from the motor cortex of control, CBD, and PSP subjects, and from the prefrontal cortex of AD subjects. Further subregional specification within these areas was not feasible due to the nature of tissue sampling. Bound antibodies were visualized using a peroxidase-polymer-based detection method with a Histofine Simple Stain MAX-PO kit (Nichirei Biosciences) and diaminobenzidine as the chromogen. Sections were counterstained with Mayer’s haematoxylin. A three-point semiquantitative analysis of neuronal immunoreactivity for PPA1 and PREP was performed using PPA1- and PREP-immunostained sections, respectively. All images were acquired using an Olympus BX53 microscope (Olympus) under identical conditions and are shown in Supplementary Figure 8. Staining intensity was categorized into three levels: + (comparable to the most intensely stained control subject), ↓ (weaker than the most intensely stained control), and ↓↓ (no or faint staining). In addition, quantitative image analysis of the IHC-positive area was performed using Fiji/ImageJ software (NIH) on all images shown in Supplementary Table 8. All images were acquired at a resolution of 4,096 × 3,000 pixels. After channel splitting, the blue channel was selected for quantification. A manual threshold was defined for each antibody and consistently applied to all images obtained under the same staining conditions. The images were then converted to binary masks, and the percentage of IHC-positive area per field was calculated.

### Statistical analysis

Perseus software (v2.1.3.0)^2^ (Tyanova et al., 2016) was used for downstream analyses of proteins (Supplementary Table 2) quantified with DIA-NN software (version 1.9.2) (Demichev et al., 2020). Specifically, one-way analysis of variance (ANOVA) across the four groups and hierarchical clustering were performed using Perseus. For multiple testing correction, permutation-based false discovery rate (FDR) correction was applied within Perseus using its default implementation for ANOVA-based comparisons, thereby controlling for type I error across large-scale proteomic datasets. FDR-adjusted *P*-values (*P*_*FDR*_) were used to define significance thresholds for proteomic comparisons. Additional statistical analyses, including the Kruskal–Wallis test for comparisons among the four groups (followed by Dunn’s post hoc test when significant), Pearson’s correlation analysis, principal component analysis (PCA), and Fisher’s exact test, were conducted using GraphPad Prism (v.10.6.1)^3^ or R (version 4.3.0).^4^ Analyses of the snRNA-seq dataset (Supplementary Table 7) were performed using Seurat software (v5.0.1)^5^ (Hao et al., 2024). Statistical significance was defined as a *P* < 0.05.

## Results

### Detection of tau protein in total homogenates from human autopsy brains by MS

**FIGURE 1:**
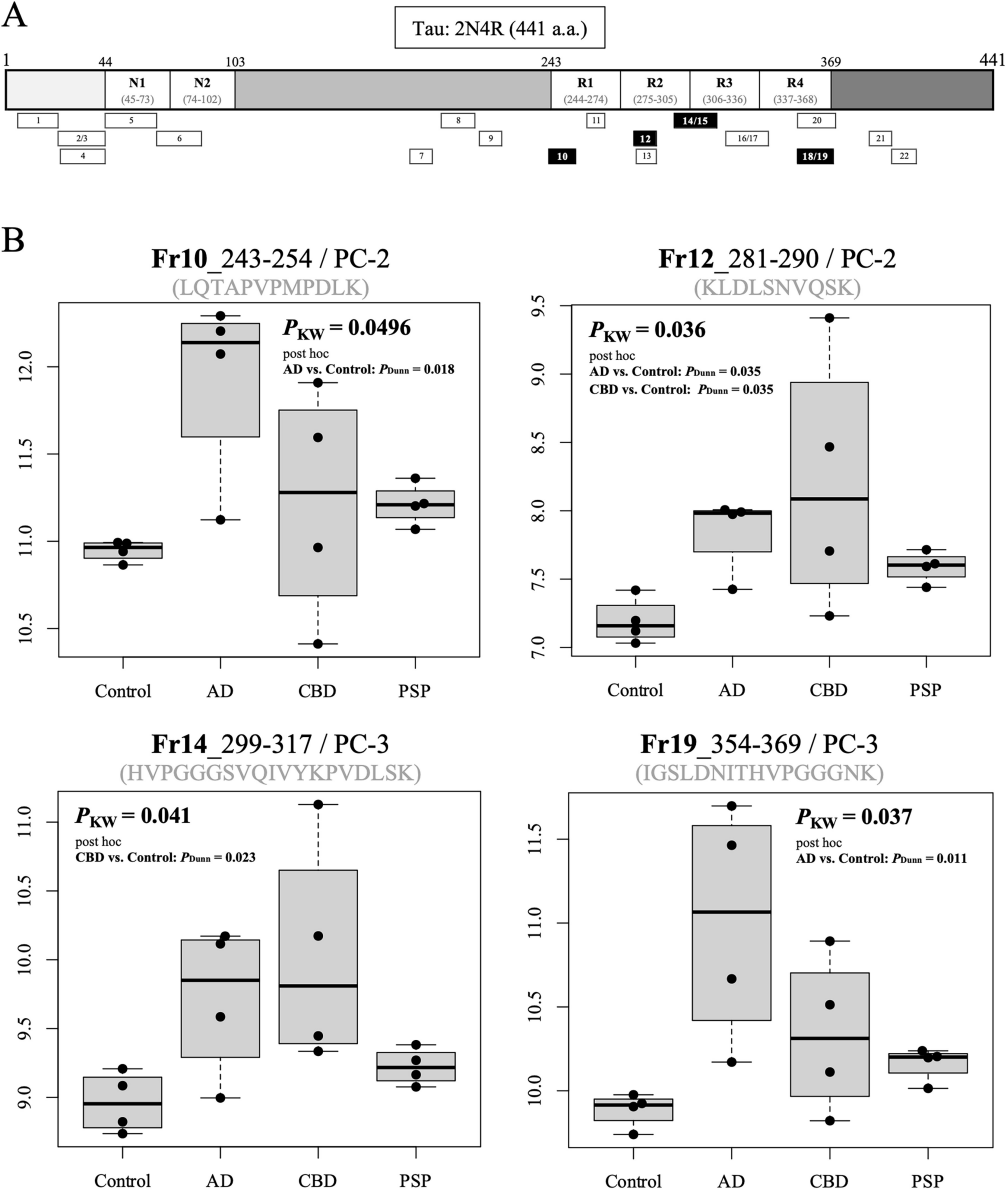
Tau fragments detected by MS. **(A)** Identified tau fragments were mapped onto the 2N4R isoform (441 a.a.). Fragment IDs (1–22) are indicated within the rectangles. Black-filled rectangles denote fragments showing significant differences in the four-group comparison. Quantitative values for each fragment are provided in Supplementary Table 3. **(B)** Box-and-whisker plots of the four fragments with significant differences: Fr10, Fr12, Fr14, and Fr19. Only statistically significant pairwise comparisons are shown with corresponding *P*-values. The y-axis values represent log2-transformed data. a.a., amino acid; Fr, fragment; *P*_*Dunn*_, *P*-value calculated using Dunn’s post hoc test; *P*_*KW*_, *P*-value calculated using the Kruskal–Wallis (KW) test; PC, precursor charge.

To identify tauopathy-associated proteins, MS was performed on total homogenates prepared from postmortem brains of 16 individuals: four cases each of CBD and PSP, which are primary tauopathies; four cases of AD, a secondary tauopathy; and four controls (Supplementary Table 1). Prior to MS, the presence of tau protein in the prepared total homogenates was examined by WB using the JESS system. We confirmed that tau protein was robustly present in all total homogenate samples (Supplementary Figure 2). MS was then performed to determine whether tau-derived fragments could be detected. In total, 22 tau-derived fragments were identified (Figure 1A and Supplementary Table 3). Comparative analyses across the four groups identified four fragments with statistically significant differences (Figure 1B): Fr10, Fr12, Fr14, and Fr19, all mapping to the microtubule-binding repeat region. For all these fragments, the tauopathy groups (AD, CBD, and PSP) tended to exhibit higher levels compared with the control group.

### Identification of tauopathy-associated proteins

**FIGURE 2:**
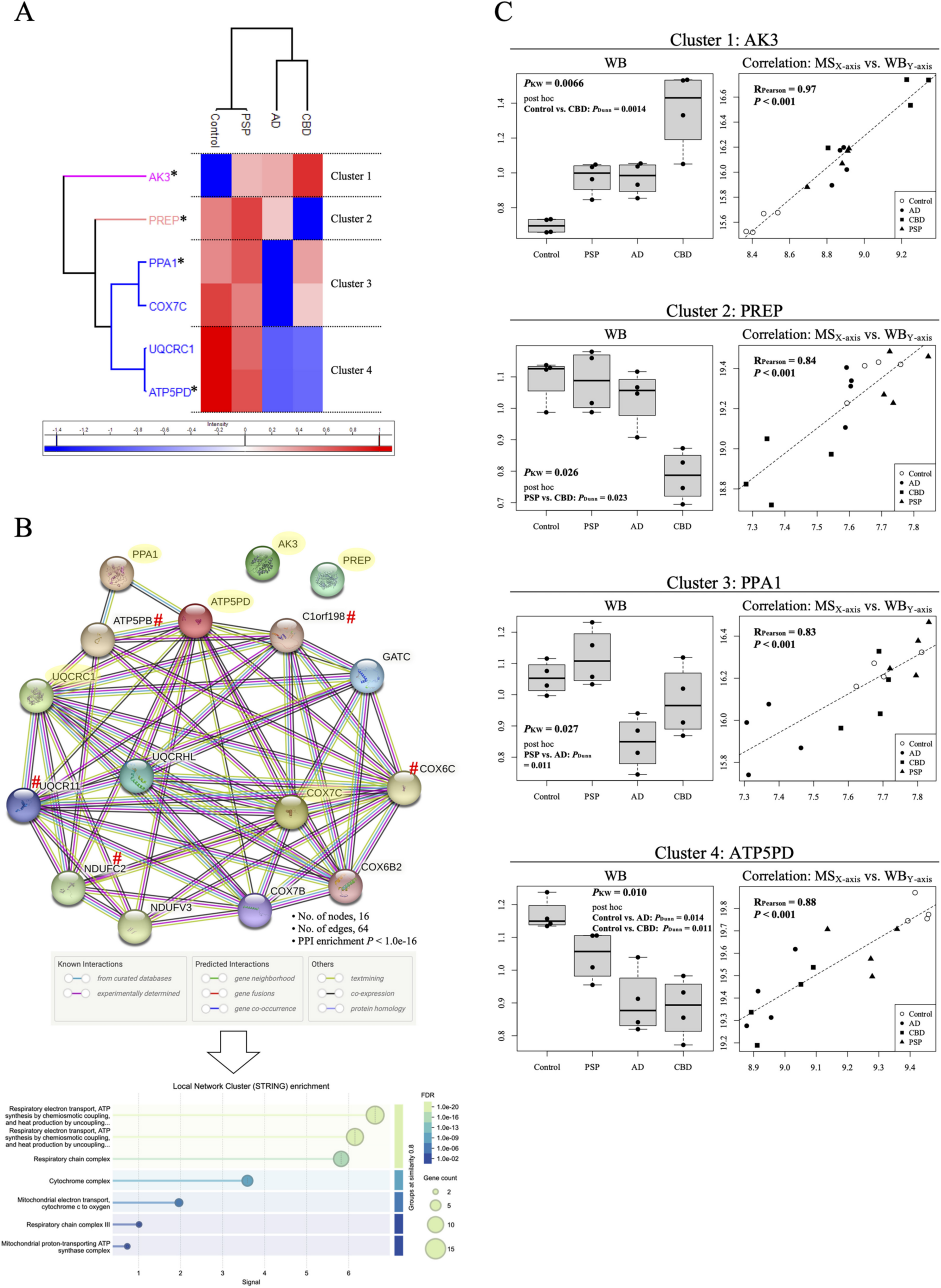
Identification of proteins associated with tauopathies. **(A)** Hierarchically clustered heatmap based on six proteins showing significant differences in the four-group comparison (ANOVA *P*_*FDR*_ < 0.005; Supplementary Tables 4, 5). The 16 subjects (Supplementary Table 1) were classified into four diagnostic groups, AD, CBD, Control, and PSP. The six proteins were divided into four clusters: Clusters 1 (AK3), 2 (PREP), 3 (PPA1 and COX7C), and 4 (UQCRC1 and ATP5PD). One representative protein from each cluster (asterisked) was selected for subsequent WB validation (Figure 2C and Supplementary Figure 5). **(B)** PPI network of MS-identified tauopathy-related proteins generated using the STRING database (https://string-db.org). Proteins highlighted in light yellow represent those detected in the MS analysis (ANOVA *P*_*FDR*_ < 0.005): ATP5PD, PPA1, COX7C, UQCRC1, AK3, and PREP (Supplementary Tables 4, 5). The network was constructed with a high-confidence interaction score (0.700) and a maximum of 10 first-shell interactors. Proteins marked with a red hash (#) are also listed in Supplementary Table 5: ATP5PB, C1orf198, COX6C, NDUFC2, and UQCR11. Enriched biological processes are shown as Local Network Cluster (STRING) enrichment (Supplementary Table 6). **(C)** Reproducibility of MS findings was assessed by JESS-based WB for AK3 (Cluster 1), PREP (Cluster 2), PPA1 (Cluster 3), and ATP5PD (Cluster 4). Box-and-whisker plots (left) show significant differences among the four groups (*P*_*KW*_ < 0.05). Dunn’s *post-hoc* test was applied to all pairwise comparisons, and only significant *P*-values are shown. WB quantification strongly correlated with MS-derived abundance for each protein (right). For the WB quantification (left), JESS-based WB signals were normalized to total protein levels, log2-transformed, and scaled to the mean value across all subjects (*n* = 16) for relative comparison among groups. In contrast, for the correlation analysis (right), JESS-based WB values were normalized to total protein levels, log2-transformed, and directly compared with the corresponding log2-transformed MS-derived protein abundance values.

We investigated whether proteins comprehensively detected by MS were associated with tauopathies. Summary data for proteins detected in each total homogenate are provided in Supplementary Table 2. A total of 3,302 proteins commonly detected across all 16 homogenates were included in the comparative analysis among the four diagnostic groups (ANOVA). The numbers of proteins with *P*_*FDR*_ less than 0.05, 0.01, 0.005, and 0.001 were 859, 114, 6, and 1, respectively (Supplementary Table 4). The full list of the 859 proteins with *P*_*FDR*_ < 0.05 is provided in Supplementary Table 5. PCA based on the 114 proteins showing *P*_*FDR*_ < 0.01 demonstrated clear separation of all 16 subjects according to diagnostic category (Supplementary Figure 3), supporting the presence of disease-specific proteomic signatures. Among the more stringently filtered proteins, hierarchical clustering analysis using the six proteins with *P*_*FDR*_ < 0.005 clearly distinguished the diagnostic groups (Figure 2A and Supplementary Figure 4). Given that validation of all 114 proteins by JESS-based WB would not be experimentally feasible, and to ensure both statistical stringency and practical tractability for downstream validation, we selected these six proteins for subsequent analyses: ATP5PD, PPA1, COX7C, UQCRC1, AK3, and PREP.

### PPI network including the tauopathy-associated proteins identified in the MS analysis

We sought to characterize the functional PPI network involving the six tauopathy-related proteins, ATP5PD, PPA1, COX7C, UQCRC1, AK3, and PREP. The STRING database (Szklarczyk et al., 2023) was used to construct the network with a high-confidence interaction score threshold of 0.700. All six proteins formed a highly interconnected network (Figure 2B). Functional enrichment analysis indicated that these proteins are predominantly associated with mitochondrial pathways, particularly the inner mitochondrial membrane protein complex, respiratory electron transport, and ATP synthesis coupled to oxidative phosphorylation (Supplementary Table 6). Enriched biological processes included oxidative phosphorylation, electron transport chain activity, thermogenesis, and mitochondrial energy metabolism (Supplementary Table 6). In addition, the network showed significant enrichment for pathways implicated in neurodegenerative disorders, including AD, Parkinson’s disease, Huntington’s disease, prion disease, and amyotrophic lateral sclerosis (Supplementary Table 6). Collectively, these findings suggest that the tauopathy-related proteins identified in this study are functionally interconnected through mitochondrial bioenergetic pathways.

### Validation of the reproducibility of MS-identified proteins

To evaluate the reproducibility of the protein levels of the six identified proteins (Figure 2 and Supplementary Table 5), we performed JESS-based WB (Supplementary Figure 5) and assessed the correlation between the WB and MS measurements. Among the six proteins, four (AK3, PREP, PPA1, and ATP5PD) showed a statistically significant and strong correlation (Pearson’s *R* > 0.8 and *P* < 0.001; Figure 2C). Accordingly, further analyses focused on these four proteins.

### Cell type-specific gene expression profiles of genes encoding the identified tau-related proteins

**FIGURE 3:**
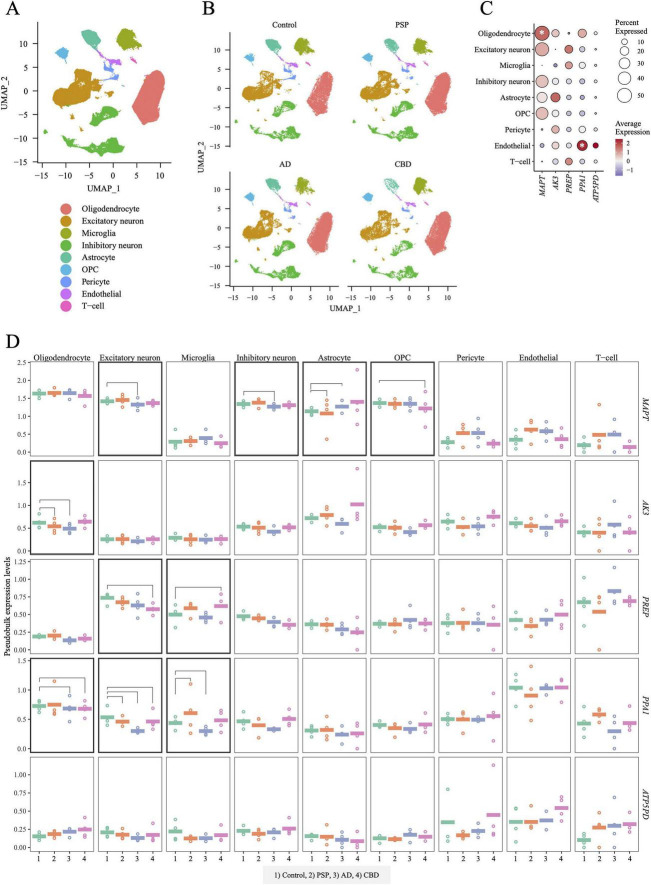
Cell-type-specific expression of four MS-identified genes in the human brain based on snRNA-seq. **(A)** UMAP plot showing clustering and cell type annotation of nuclei profiled from 16 subjects (Supplementary Table 1). Nine major cell types were identified: oligodendrocytes, excitatory neurons, microglia, inhibitory neurons, astrocytes, OPCs, pericytes, endothelial cells, and T cells. **(B)** UMAP plots stratified by diagnostic groups: Control, PSP, AD, and CBD. **(C)** Dot plot showing expression of *AK3*, *PREP*, *PPA1*, and *ATP5PD* across the nine cell types. As *MAPT*, the tau-encoding gene, is a key gene implicated in tauopathies, it was also included in this analysis. Dot size represents the percentage of nuclei expressing each gene (Percent Expressed), and color intensity indicates scaled normalized expression (Average Expression). *MAPT* and *PPA1* were highly expressed in oligodendrocytes and endothelial cells, respectively (asterisks). **(D)** Pseudo-bulk expression across cell types and diagnostic groups. Each dot represents one individual, generated by aggregating single-nucleus counts per subject. Mean expression across four biological replicates is shown by colored bars. Black horizontal lines indicate significant differences (*P* < 0.01) between each diagnostic group (PSP, AD, or CBD) and controls. ATP5PD exhibited no significant differences across cell types.

To clarify the cell type-specific expression of genes encoding the four identified tau-related proteins (AK3, PREP, PPA1, and ATP5PD), we conducted snRNA-seq on the same set of 16 subjects (Supplementary Table 1) used for the MS analysis. Summary statistics for this analysis are provided in Supplementary Table 7 and Supplementary Figure 6. Nine major cell types were identified, listed below in alphabetical order: astrocytes, endothelial cells, excitatory neurons, inhibitory neurons, microglia, oligodendrocyte precursor cells (OPCs), oligodendrocytes, pericytes, and T cells (Figures 3A,B). In addition to the four tauopathy-related genes (*AK3*, *PREP*, *PPA1*, and *ATP5PD*), we examined the gene expression levels of the key tauopathy gene *MAPT* across nine cell populations. *MAPT* showed higher expression in oligodendrocytes, whereas *PPA1* was highly expressed in endothelial cells (Figure 3C). The other three genes, *AK3*, *PREP*, and *ATP5PD*, did not exhibit clear cell-type-specific expression (Figure 3C). For each of the nine classified cell populations, expression levels of *MAPT* and the four MS-identified genes, *AK3*, *PREP*, *PPA1*, and *ATP5PD*, were compared between the control and tauopathy groups. Significant differences were observed for all genes except *ATP5PD* in at least one cell population (Figure 3D): excitatory neurons, inhibitory neurons, astrocytes, and OPCs in *MAPT*; oligodendrocytes in *AK3*; excitatory neurons, and microglia in *PREP*; and oligodendrocytes, excitatory neurons, and microglia in *PPA1*. We generated pseudo-bulk expression profiles by aggregating snRNA-seq data across the nine cell populations and examined their correlations with corresponding protein abundance (Supplementary Figure 7). Only *PREP* showed a statistically significant correlation between pseudo-bulk RNA expression and JESS-based WB quantification (Supplementary Figure 7B): *P* = 0.016, and Pearson’s *R* = 0.59.

### IHC of PPA1 and PREP in human autopsy brains

**FIGURE 4:**
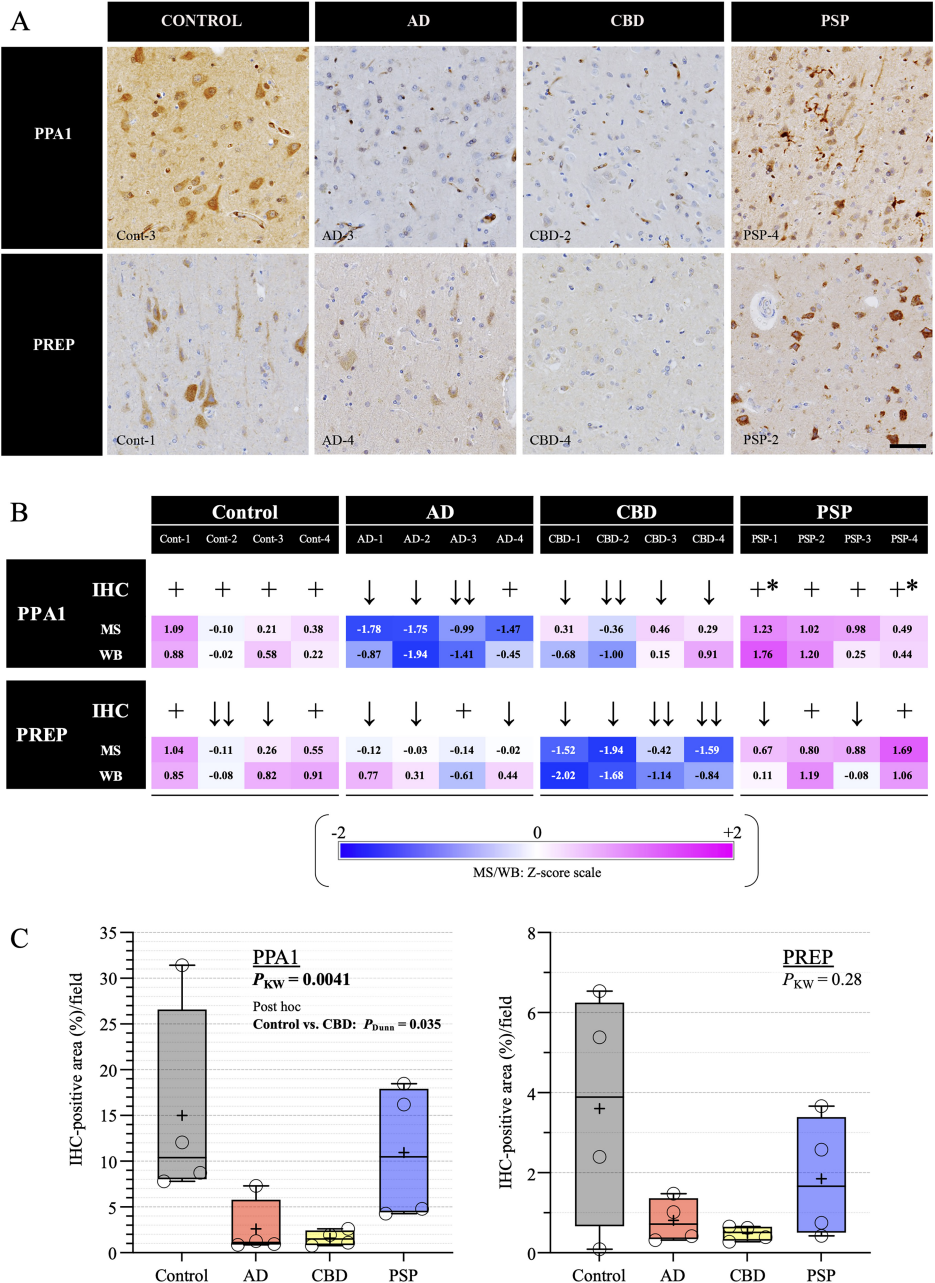
Expression patterns of PPA1 and PREP in tauopathy brains. **(A)** Representative immunohistochemical images of PPA1 and PREP in frontal cortex sections from subjects with AD and in motor cortex sections from those with CBD, PSP, and controls. In control brains, both proteins are predominantly expressed in neurons. Notably, increased PPA1 immunoreactivity in microglia was observed in two PSP subjects (PSP-1 and PSP-4; asterisks in **B**), although only PSP-4 is shown here. Scale bar = 50 μm. **(B)** Semiquantitative evaluation of neuronal immunoreactivity for PPA1 and PREP in AD, CBD, PSP, and control subjects. IHC results are shown alongside Z-score-normalized MS values and densitometric quantification from JESS-based WB. The semiquantitative trends were largely consistent with the MS and WB findings. **(C)** Quantitative analysis of IHC-positive area (%) for PPA1 (left) and PREP (right) across all subjects (*n* = 16), corresponding to the data shown in Supplementary Figure 8. Each open circle indicates an individual subject, and “+” denotes the mean. Group comparisons were performed using the Kruskal–Wallis test, followed by Dunn’s post hoc test when appropriate to compare AD, CBD, and PSP with controls (showing only statistically significant *P*-values). Bold indicates statistical significance. When interpreted together with the semiquantitative results in **(B)**, both PPA1 and PREP tend to be reduced in AD and CBD compared with controls and PSP.

Based on the integrated findings from the MS, JESS-based WB, and snRNA-seq analyses, we selected PPA1 and PREP for further in situ validation. Both genes exhibited significant disease-associated alterations across multiple cell populations in the snRNA-seq analysis (Figure 3). We therefore performed IHC to examine their spatial distribution in brain tissue (Figure 4). The same set of 16 subjects was analyzed (Supplementary Table 1). In control brains, both PPA1 and PREP were primarily localized to neurons (Figure 4A). Notably, prominent PPA1 immunoreactivity was observed in a subset of microglia in sections from two PSP patients (PSP-1 and PSP-4) (Figures 4A,B). Semiquantitative assessment of PPA1 and PREP staining was generally consistent with the findings from MS and JESS-based WB analyses (Figure 4B). Additional quantitative image analysis further supported these findings, showing a trend toward reduced PPA1 and PREP immunoreactivity in AD and CBD compared with controls and PSP (Figure 4C and Supplementary Figure 8).

## Discussion

Through the MS analysis of postmortem brain tissues (total homogenates) from primary tauopathies, CBD and PSP, as well as the secondary tauopathy AD (Supplementary Table 1), we identified multiple proteins associated with tauopathies (Figure 2A and Supplementary Table 5). Among these, we focused our subsequent analyses on six proteins showing ANOVA *P*_*FDR*_ < 0.005: AK3, ATP5PD, COX7C, PPA1, PREP, and UQCRC1 (Figure 2A and Supplementary Figure 4). PPI network analysis revealed that these proteins were predominantly related to mitochondrial structure and function (Figure 2B and Supplementary Table 6). This result is largely consistent with previous proteomic evidence of mitochondrial dysfunction in tauopathies and further supports a role for mitochondrial pathways in tauopathy pathogenesis (Nementzik et al., 2025).

Mitochondria are ubiquitous organelles present in virtually all cell types and have been reported to be affected across a wide range of neurodegenerative diseases, including AD (Swerdlow et al., 2010). While our findings further support the involvement of mitochondrial alterations in tauopathies, it remains unclear whether these changes represent a primary pathogenic driver or a secondary consequence of neurodegeneration. Future mechanistic and longitudinal studies, including cell-based and animal model investigations, will be required to clarify the causal relationship between mitochondrial dysfunction and tau-mediated pathology. In this context, given the limited number of MS-based studies using postmortem brains from rare tauopathies such as CBD and PSP (Johnson et al., 2024; Kavanagh et al., 2025; Lantero-Rodriguez et al., 2024; Nementzik et al., 2025), the catalog of tauopathy-associated proteins identified in the present study (Supplementary Table 5) represents a valuable resource for elucidating disease mechanisms and informing future therapeutic development.

Notably, prominent PPA1 immunoreactivity was observed in a subset of microglia in PSP brains (Figure 4). Microglia are resident innate immune cells of the central nervous system that play a crucial role in neuroinflammation, and their activation is closely associated with metabolic reprogramming and mitochondrial alterations. PPA1 is an energy-metabolizing enzyme implicated in mitochondrial maintenance (Wang et al., 2022). Experimental inflammatory models further support this link. Lipopolysaccharide (LPS), a bacterial endotoxin that activates innate immune signaling, and interferon gamma (IFNγ), a pro-inflammatory cytokine involved in immune priming, are widely used to induce microglial activation *in vitro*. In this context, a recent transcriptomic analysis demonstrated that *PPA1* expression is significantly upregulated in human iPSC-derived microglia under combined LPS and IFNγ stimulation (Heiss et al., 2026). Given that PSP is a primary tauopathy, accumulation of hyperphosphorylated tau may trigger microglial activation accompanied by metabolic reprogramming and increased PPA1 expression. Thus, PPA1 induction in PSP microglia may reflect altered energy metabolism or inflammatory activation. However, whether this represents a downstream consequence of tau pathology or a compensatory metabolic adaptation remains to be clarified.

Although this study integrates proteomic and transcriptomic approaches, the primary focus was on proteomic alterations identified by MS, with snRNA-seq used to provide complementary insight into the cellular context of the identified proteins rather than direct RNA-protein correspondence. Consistent with this approach, we observed limited concordance between transcriptomic and proteomic data, with only PREP showing a significant correlation between pseudo-bulk RNA expression and protein abundance (Supplementary Figure 7B). This discrepancy is not unexpected, as protein levels are influenced by post-transcriptional regulation and may not directly reflect RNA expression. In addition, differences in resolution between bulk proteomics and single-nucleus transcriptomics may contribute to discordance. Notably, snRNA-seq revealed cell type-specific expression patterns, such as enrichment of PPA1 in endothelial cells (Figure 3C), supporting the interpretation that the observed proteomic alterations may originate from distinct cellular sources. Collectively, these findings support the complementary nature of proteomic and transcriptomic approaches, and future studies incorporating cell type-resolved multi-omics will be important to further clarify the underlying mechanisms.

In this study, several limitations should be acknowledged. First, the sample size was modest, with only four subjects per diagnostic group, limiting statistical power and generalizability. Second, only a single brain region per individual was analyzed (frontal cortex: prefrontal cortex for AD and motor cortex for CBD, PSP, and controls), preventing assessment of region-specific proteomic heterogeneity across tauopathies. Moreover, the use of different brain regions across diagnostic groups may introduce additional variability and could confound direct comparisons of proteomic profiles between groups. Third, proteomic analysis was restricted to total homogenates from the specified cortical region, which, while providing a broad and unbiased overview of protein expression, introduces several important limitations. This approach mixes proteins derived from multiple cell types, thereby obscuring cell type–specific contributions, and does not distinguish between soluble and insoluble fractions, the latter of which are particularly relevant to tau aggregation and pathology. In addition, low-abundance proteins may be diluted and remain undetected in such bulk analyses. Finally, no independent validation cohort or two-stage design was included to confirm the reproducibility of the identified tauopathy-associated protein signatures. Future studies incorporating larger and anatomically diverse cohorts, as well as fraction- and cell type–resolved proteomics and independent validation, will be important to further validate and extend these findings.

Taken together, our findings suggest that mitochondria-associated proteomic alterations may be present across primary and secondary tauopathies. However, these observations are hypothesis-generating and require further investigation to clarify their mechanistic significance, particularly through future studies addressing the limitations of the present work.

## Data Availability

The datasets presented in this study can be found in online repositories. The names of the repository/repositories and accession number(s) can be found at: http://www.proteomexchange.org/, PXD072806; https://repository.jpostdb.org/, JPST004307; https://www.ncbi.nlm.nih.gov/geo/, GSE317746.

## Footnotes

1
https://string-db.org

2
https://maxquant.net/perseus/

3
https://www.graphpad.com

4
https://www.r-project.org

5
https://satijalab.org/seurat/

## Supplementary material

Supplementary Figure 1 Analytical workflow of the study. A total of 12 tauopathy autopsy brains (four each from AD, CBD, and PSP) and four control brains were included in the analysis (Supplementary Table 1). Each step of the workflow is accompanied by corresponding figure and table numbers (shown in blue). Representative immunohistochemical images of phosphorylated tau stained with AT8 are shown for one subject each with AD, CBD, PSP, and a control (AD: frontal cortex; CBD, PSP, and control: motor cortex). White arrows indicate neurofibrillary tangles (AD), black arrows indicate astrocytic plaques (CBD), and black arrowheads indicate tufted astrocytes (PSP). Antibodies used for IHC are listed in Supplementary Table 9. Scale bar = 50 μm for all images. LC, liquid chromatography; Ref, reference; SF, supplementary figure; ST, supplementary table.

Supplementary Figure 2 Confirmation of tau protein in total homogenates. **(A)** WB analysis of tau in total homogenates from autopsied brains using the JESS system with the primary anti-tau antibody T46 (Supplementary Table 9). **(B)** Total protein staining of the electrophoresed homogenates shown in **(A)**. **(C)** Box-and-whisker plot showing tau abundance quantified by MS. The y-axis values represent log2-transformed data. Detailed statistics are provided in Supplementary Table 5 (#847). Comparison across the four diagnostic groups revealed a significant difference (ANOVA *P*_*FDR*_ = 0.049). Cont, control; *P*_*FDR*_, *P*-value adjusted for multiple testing using the false discovery rate (FDR) method implemented in Perseus (PMID: 27348712) with default permutation-based settings.

Supplementary Figure 3 Principal component analysis of the 16 subjects. **(A)** Principal component analysis based on 114 proteins showing significant differences across the four diagnostic groups (ANOVA *P*_*FDR*_ < 0.01; Supplementary Tables 4, 5). The first and second principal components clearly separated AD, CBD, Control, and PSP subjects. **(B)** Bar plot showing variance explained by the first 15 principal components. In **(A)**, subjects are plotted using Principal Component (PC) 1 (74.5%) and PC2 (11.2%). Dim, dimension.

Supplementary Figure 4 Subject-level hierarchical clustering heatmap of the six identified proteins. Proteins marked with an asterisk (*), AK3, PREP, PPA1, and ATP5PD, were selected as cluster representatives (Clusters 1–4) for reproducibility assessment by JESS-based WB (Figure 2C and Supplementary Figure 5). Cluster assignment was defined according to the disease-level clustering shown in Figure 2A.

Supplementary Figure 5 JESS-based WB validation of four MS-identified proteins: AK3 **(A)**, PREP **(B)**, PPA1 **(C)**, and ATP5PD **(D)**. WB signals are shown on the left, with corresponding total protein staining on the right. Antibody details are provided in Supplementary Table 9. Ab, antibody.

Supplementary Figure 6 Summary statistics of the snRNA-seq data. Box-and-whisker plots showing **(A)** nuclei per postmortem brain passing QC, **(B)** genes detected per QC-passed nucleus, and **(C)** UMIs detected per QC-passed nucleus. “Overall (*n* = 16)” indicates all subjects combined (Supplementary Table 1). Detailed gene and UMI statistics are provided in Supplementary Table 7. Max, maximum; Min, minimum; QC, quality control; SD, standard deviation; UMI, unique molecular identifier.

Supplementary Figure 7 Correlation between protein abundance quantified by MS and pseudo-bulk gene expression derived from snRNA-seq. The analysis focused on four MS-identified proteins: AK3 (Cluster 1), PREP (Cluster 2), PPA1 (Cluster 3), and ATP5PD (Cluster 4) (Figure 2A). In **(A)**, the Y-axis represents protein abundance quantified by MS, whereas in **(B)**, it represents abundance measured by JESS-based WB (Figure 2C). In both panels, the X-axis shows pseudo-bulk gene expression from snRNA-seq (Figure 3). A significant correlation was observed only for PREP in **(B)** (R_*Pearson*_ = 0.59, *P* = 0.016); the plot is highlighted with a bold black outline.

Supplementary Figure 8 IHC of PPA1 and PREP in all subjects (*n* = 16) included in this study. Quantitative IHC analysis was performed by calculating the percentage of IHC-positive area per field. The bold percentage values shown in the upper right corner of each image indicate the corresponding quantified positive area, which was used for the box-and-whisker plots in Figure 4C. Scale bar = 50 μm.
