# Supplementary material for: Mass spectrometry-based proteomic profiling of human tauopathy brains suggests mitochondria-associated alterations
Source: Front Mol Neurosci. 2026 May 22;19:1815858. doi: 10.3389/fnmol.2026.1815858 (PMC13236917; doi:10.3389/fnmol.2026.1815858)

Supplementary Figure 1

Provided by Dept. of Pathology, Brain Res. Inst., Niigata Univ.

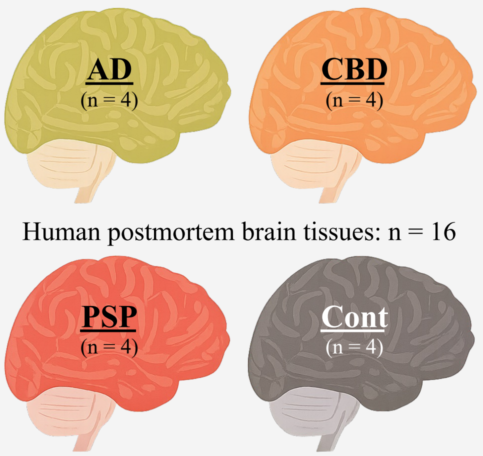

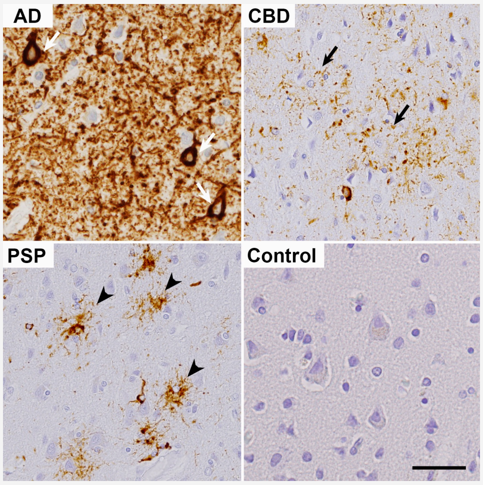

Details of each subject are provided in **ST-1**

Total homogenate preparation

- Using approx. **100 mg** of frozen frontal cortex per subject
- Based on the method described by Diner I *et al.* (2017 [PMID, 29155708])

- Confirmation of the presence of tau proteins

• **SF-2**

LC-MS/MS

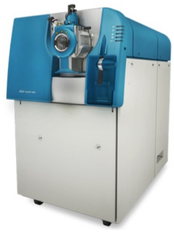

TripleTOF 5600+ system
(SCIEX)

- Total homogenates containing **100 µg** of protein per sample
- Protein precipitation with trichloroacetic acid
- Acetone washing and air-drying
- Sample pretreatment: reduction and alkylation of cysteine residues, trypsin digestion, and peptide purification
- Protein quantification using a TripleTOF 5600+ system, outsourced to the Center for Research Promotion, School of Medicine, Niigata University: https://crp.med.niigata-u.ac.jp/index

Quantification of peptides and proteins

DIA-NN
(v1.9.2)

- A high-accuracy, high-throughput tool for peptide/protein identification and quantification, reported by Demichev V *et al.* (2020 [PMID: 31768060])

Analysis of the processed and quantified data

Perseus
(v2.1.3.0)

- A platform providing extensive statistical, visualization, and biological analysis tools for MS-based proteomics, as described by Tyanova S *et al.* (2016 [PMID: 27348712])

Identification of proteins associated with tauopathies
Comparative analysis at the protein level: AD vs. CBD vs. PSP vs. $\text{Control}_{\text{Ref}}$

• **ST-4**
• **ST-5**

- Verification of the reproducibility of the MS-based quantitative data by WB

• **Figure 2**
• **SF-5**

- Analysis of cell-type–specific gene expression in human postmortem brains using snRNA-seq

• **Figure 3**
• **ST-8**

Identification of the brain distribution of the tauopathy-associated proteins
Analysis by immunohistochemical staining of human autopsy brain tissues

• **Figure 4**
• **SF-8**

Supplementary Figure 2

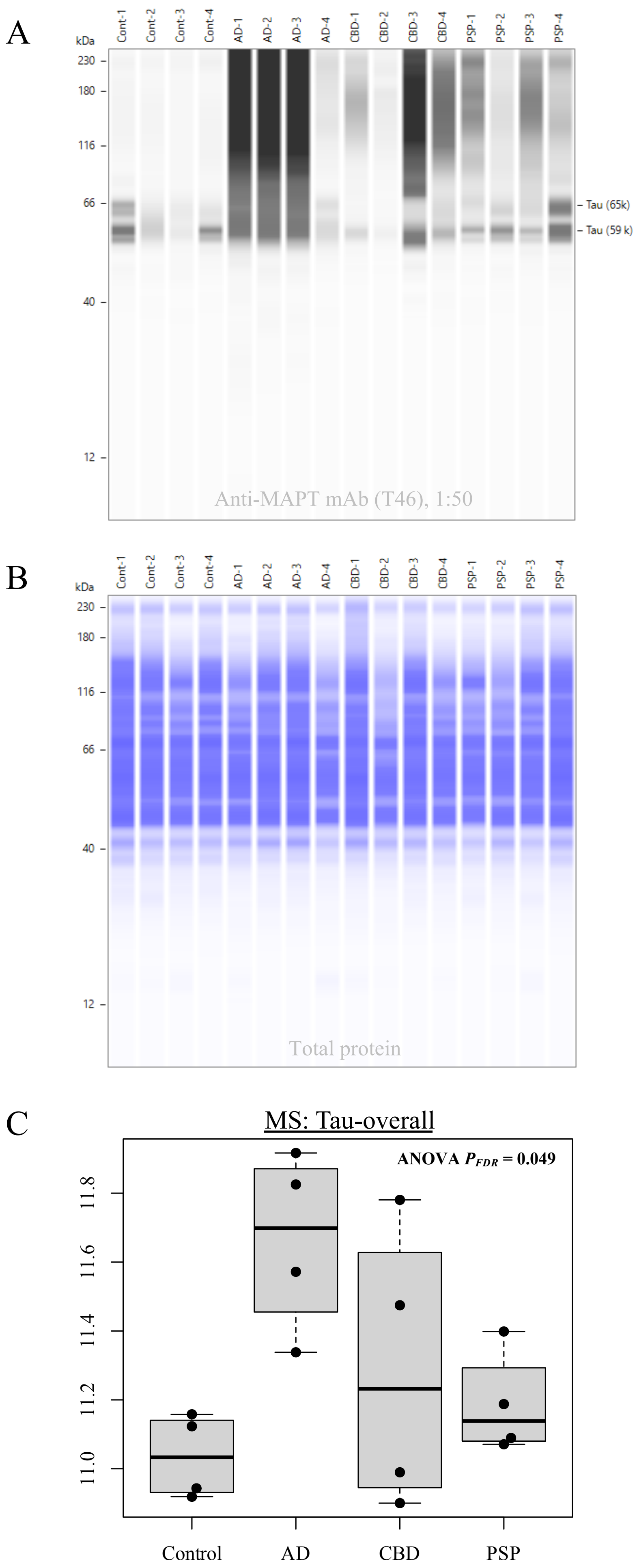

Supplementary Figure 3

A

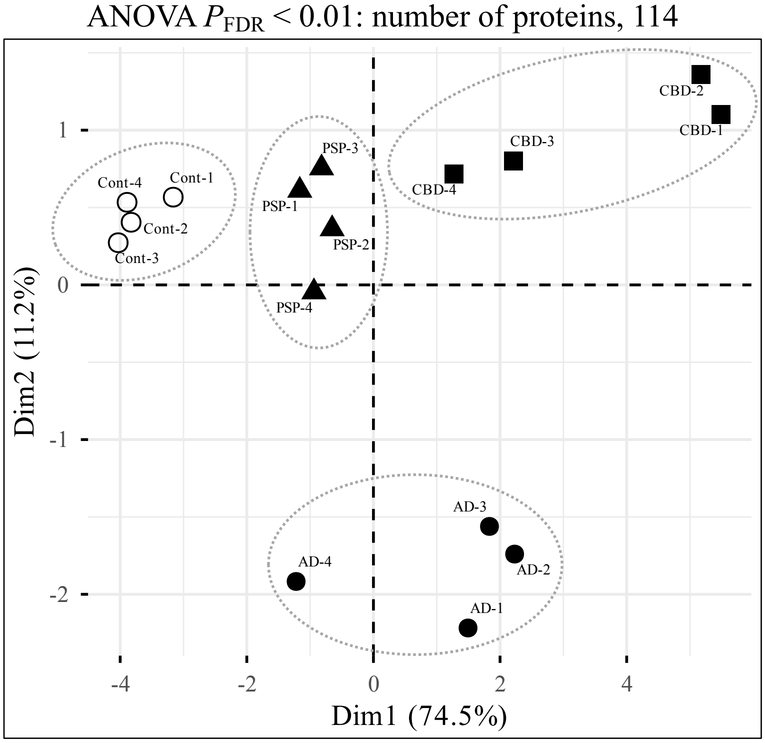

B

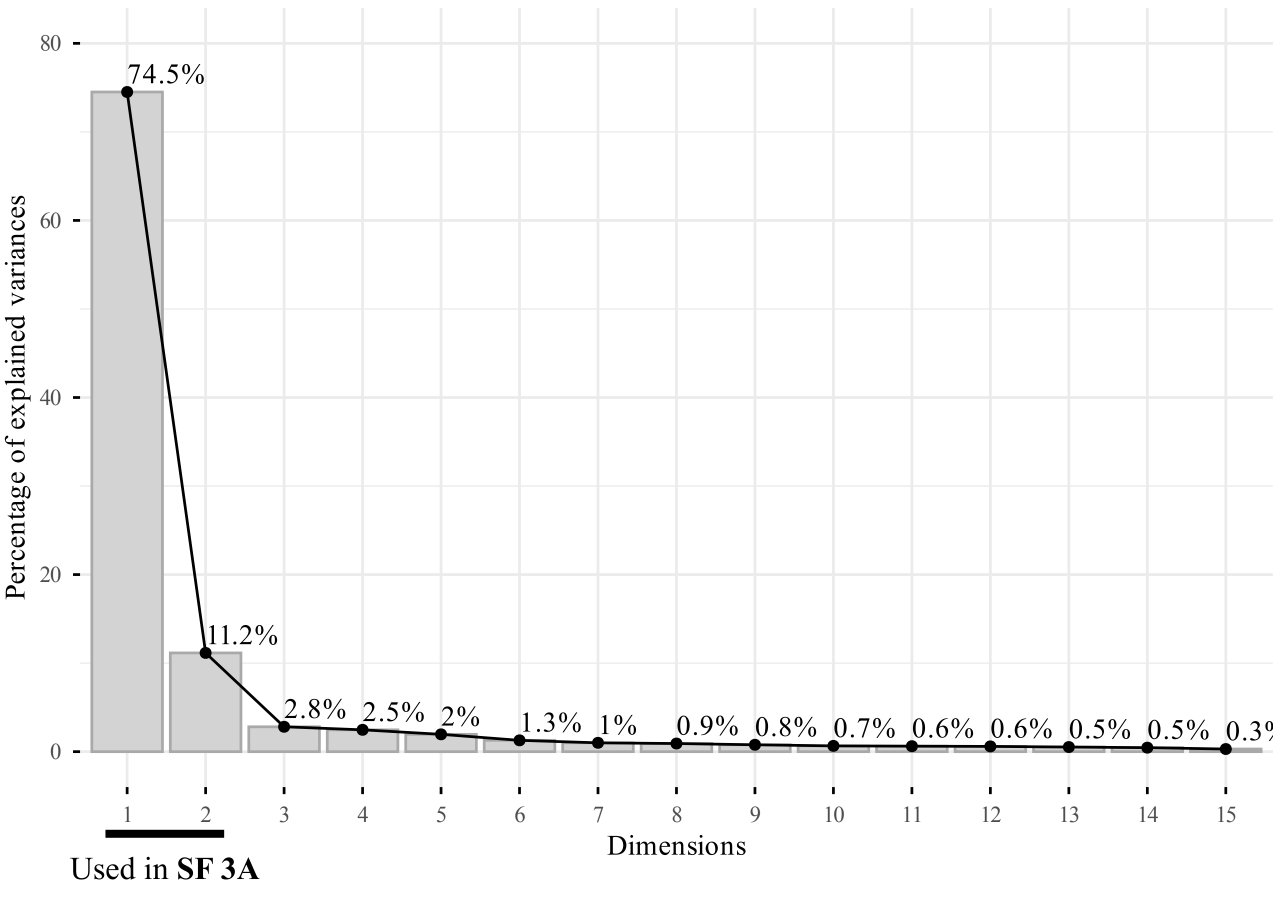

Supplementary Figure 4

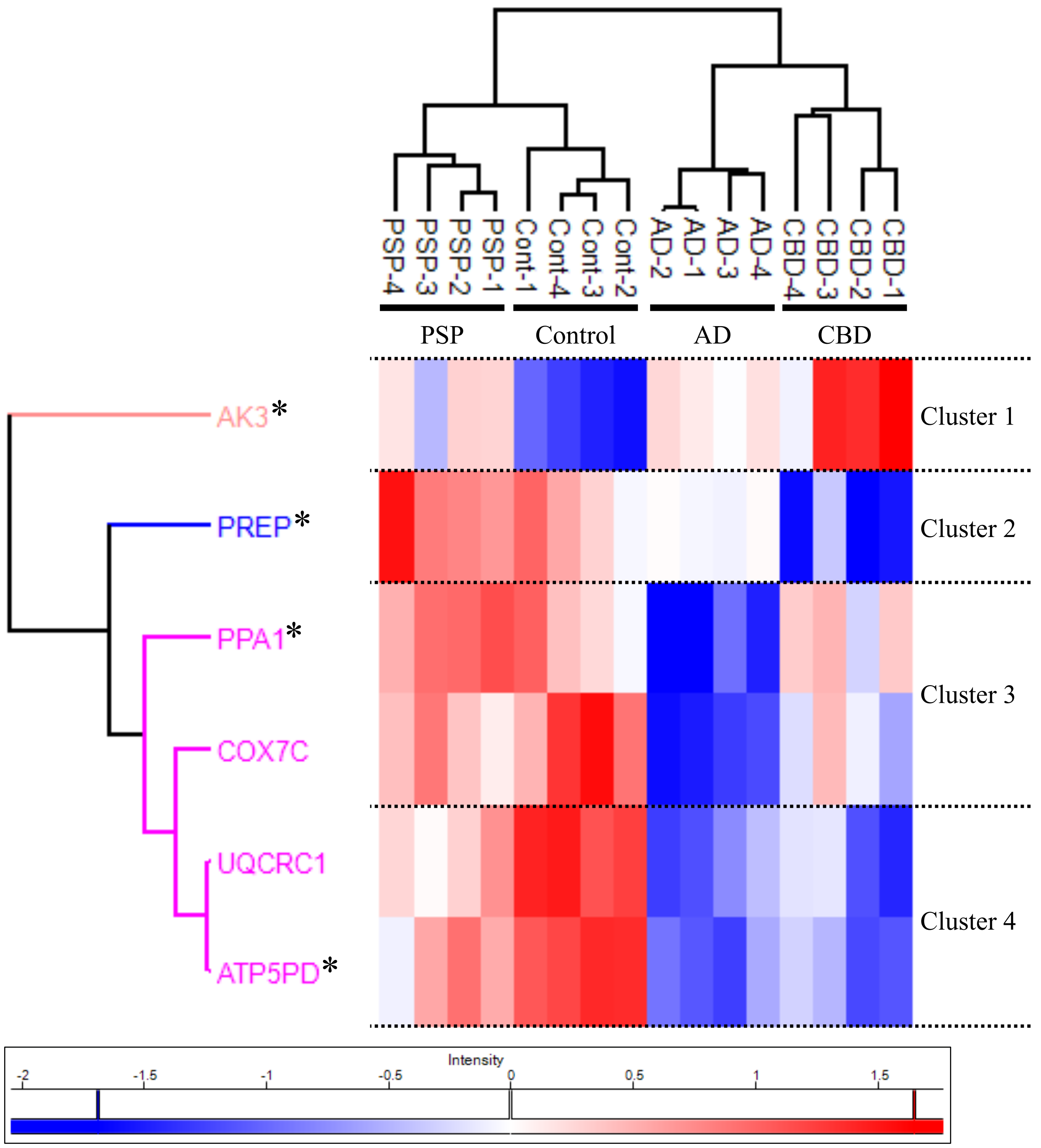

Supplementary Figure 5

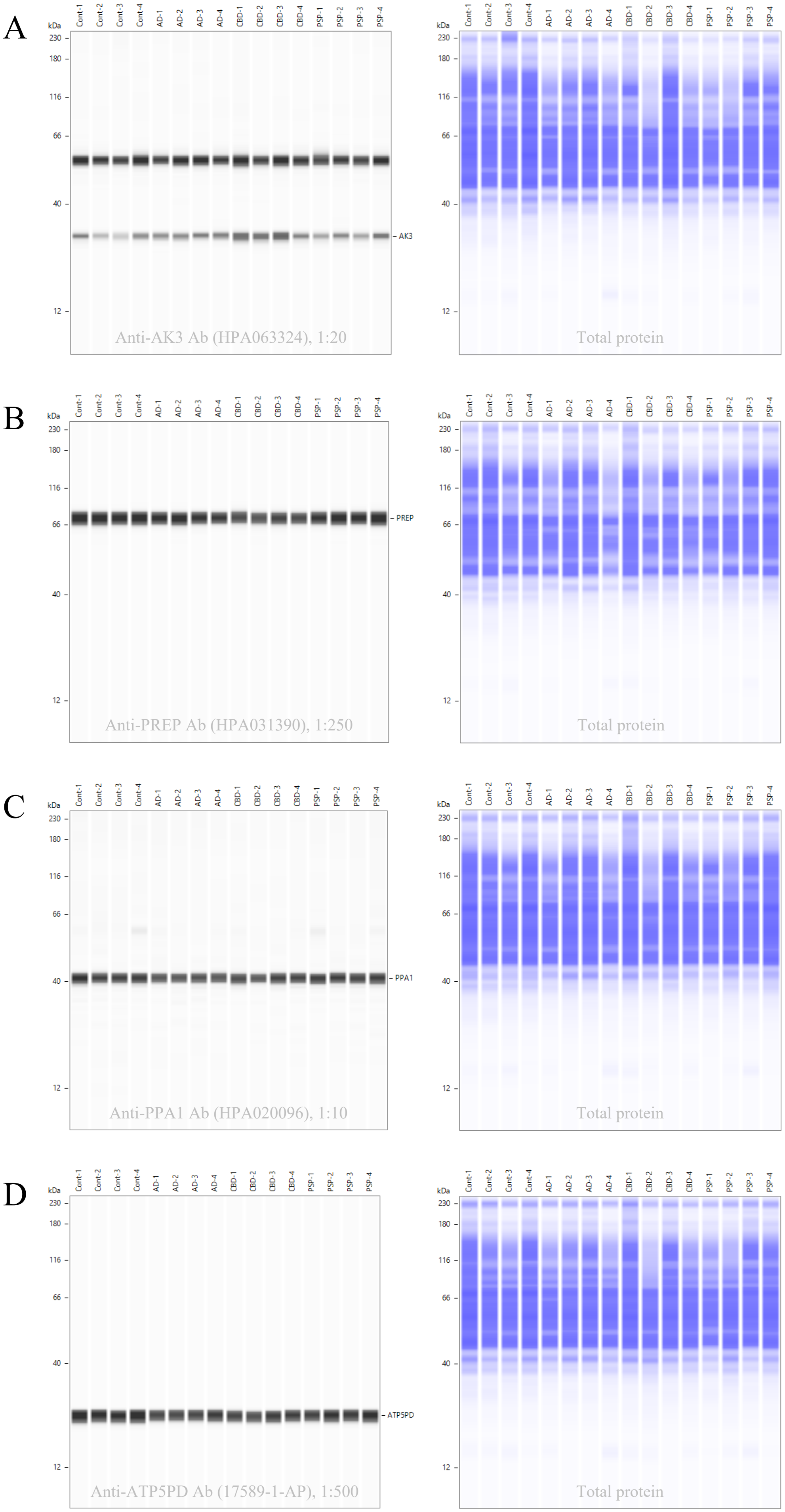

Supplementary Figure 6

A

Summary: nuclei
Overall (n = 16)

	Nuclei	
	Input	QC-passed
Mean	9,531.3	8,613.1
Median	9,894.5	9,153.0
SD	2,181.9	2,079.4
Min	4,743	3,644
Max	13,140	11,535

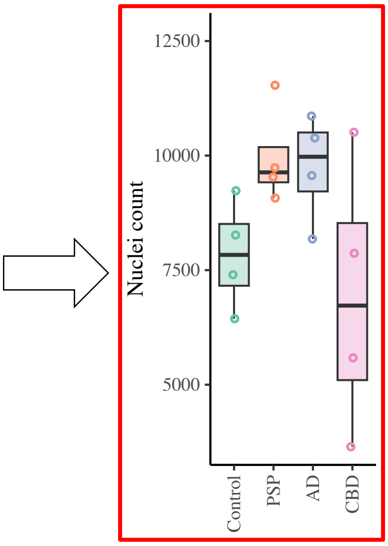

B

Summary: gene
Overall (n = 16)

	Gene
Mean	2,783.8
Median	2,216.0
SD	1,880.5
Min	275
Max	12,982

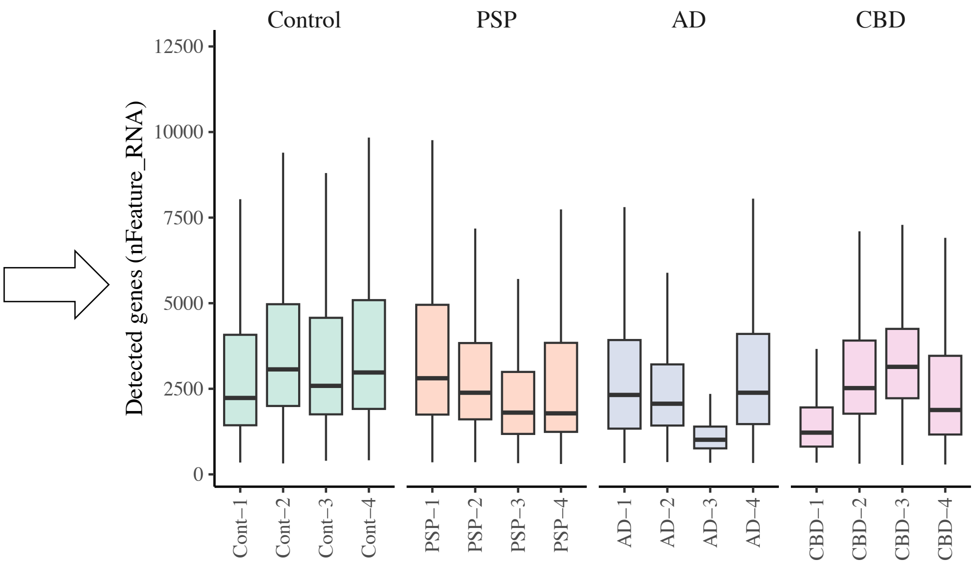

C

Summary: UMI
Overall (n = 16)

	UMI
Mean	7,934.2
Median	4,500.0
SD	9,148.6
Min	438
Max	155,886

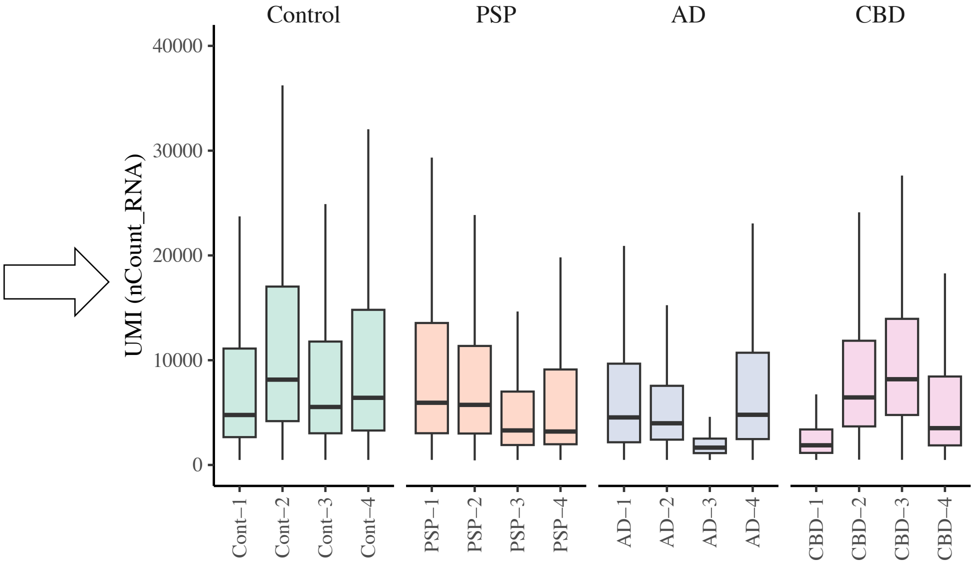

Supplementary Figure 7

A

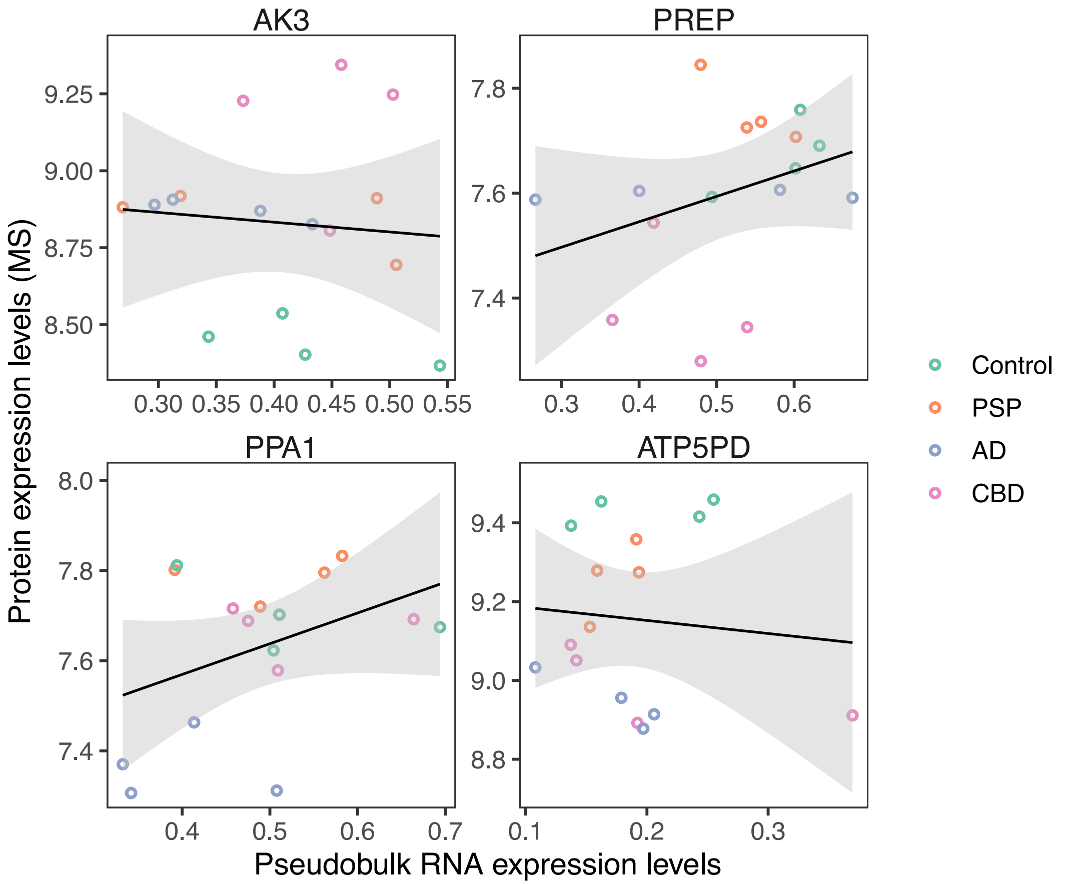

B

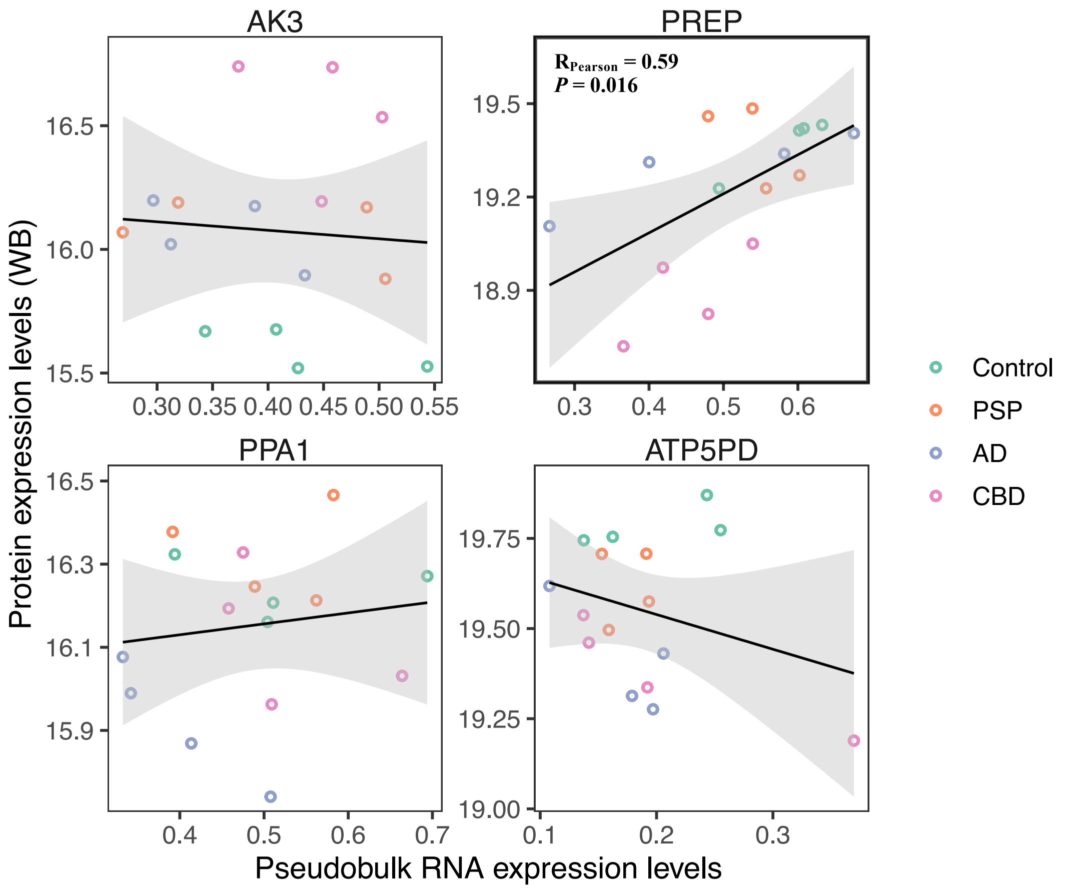

Supplementary Figure 8

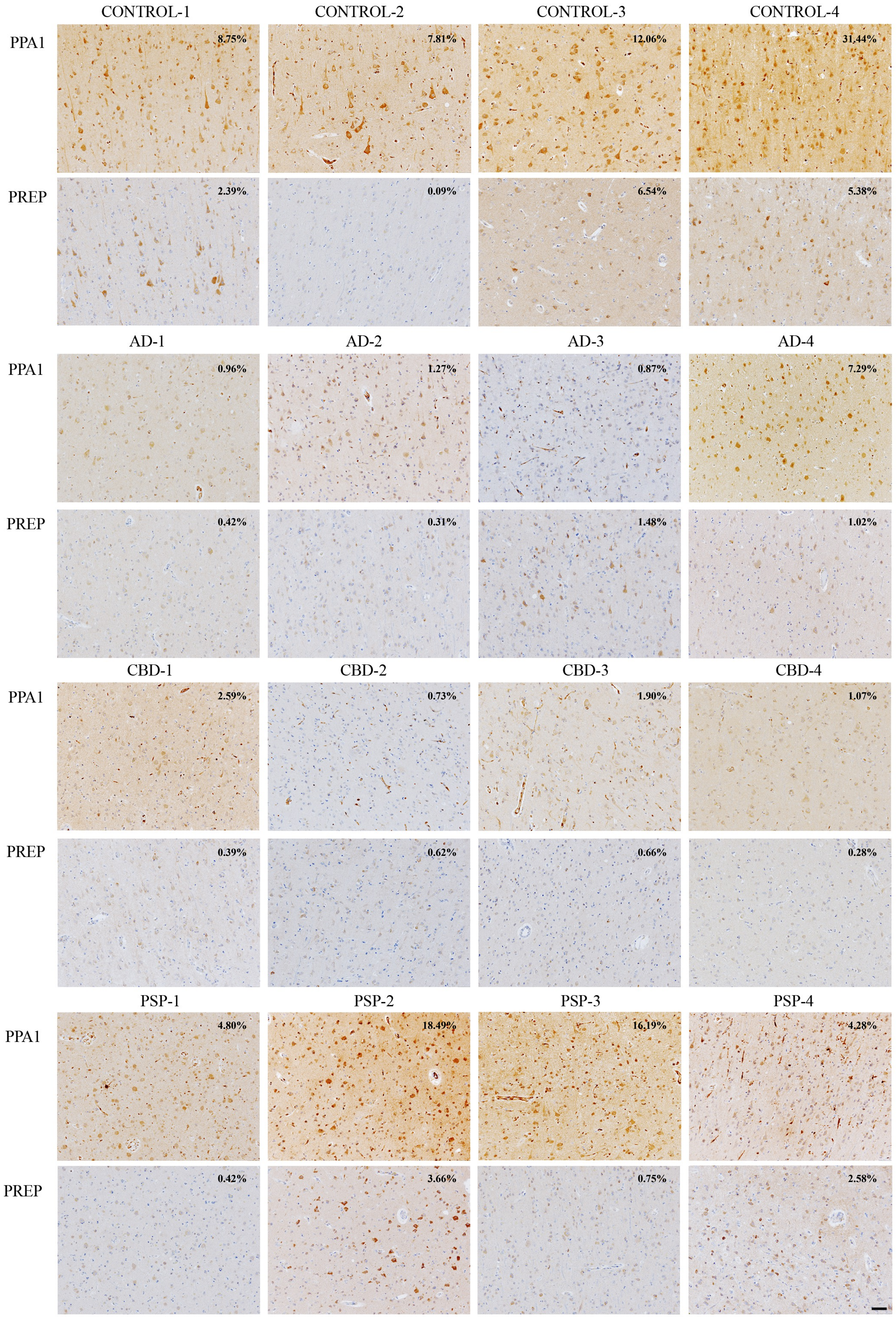

Supplement: Supplementary Figure 1 — Analytical workflow of the study. A total of 12 tauopathy autopsy brains (four each from AD, CBD, and PSP) and four control brains were included in the analysis (Supplementary Table 1). Each step of the workflow is accompanied by corresponding figure and table numbers (shown in blue). Representative immunohistochemical images of phosphorylated tau stained with AT8 are shown for one subject each with AD, CBD, PSP, and a control (AD: frontal cortex; CBD, PSP, and control: motor cortex). White arrows indicate neurofibrillary tangles (AD), black arrows indicate astrocytic plaques (CBD), and black arrowheads indicate tufted astrocytes (PSP). Antibodies used for IHC are listed in Supplementary Table 9. Scale bar = 50 μm for all images. LC, liquid chromatography; Ref, reference; SF, supplementary figure; ST, supplementary table. [file Data_Sheet_2.pdf]
